# *Aeromonas hydrophila* sepsis after ERCP and laparoscopic cholecystectomy: a case report

**DOI:** 10.3389/fmed.2026.1883706

**Published:** 2026-07-22

**Authors:** Jinli Liu, Mengting Yao, Chengxiao Liang, Xin Dai, Chao Deng, Yulong An, Minjie Yu, Ze Peng, Xinyi Zhang, Yuyun Wang, Baifa Sheng, Hao Luo

**Affiliations:** 1 Department of Hepatobiliary Surgery, The Affiliated Hospital of Southwest Medical University, Luzhou, China; 2 Department of General Surgery, The General Hospital of Western Theater Command, Chengdu, China; 3 Department of Laboratory Medicine, The General Hospital of Western Theater Command, Chengdu, China

**Keywords:** *Aeromonas hydrophila*, case report, endoscope sterilization, ERCP, sepsis

## Abstract

Endoscopic retrograde cholangiopancreatography is crucial for treating pancreaticobiliary diseases with biliary stones, but it can lead to serious complications, including severe systemic sepsis. Sepsis is a life-threatening critical condition. The 1 h bundle recommended by the Surviving Sepsis Campaign remains the core strategy for early management. Although the 2021 and 2026 Surviving Sepsis Campaign guidelines have adopted a stratified approach to antibiotic timing in non-shock patients, personalized early intervention is crucial for high-risk critically ill individuals. We present a 76-year-old female with cholecystolithiasis complicated by choledocholithiasis who developed *Aeromonas hydrophila* sepsis following Endoscopic Retrograde Cholangiopancreatography stone extraction and laparoscopic cholecystectomy. Within a span of 10 h, the patient’s white blood cell count experienced a significant decline, accompanied by increased levels of inflammatory biomarkers and a Sequential Organ Failure Assessment score of 2. The 1 h sepsis bundle was promptly implemented, including blood culture, lactate measurement, intravenous fluid resuscitation, and empirical meropenem. Blood culture identified *Aeromonas hydrophila* infection, and targeted antimicrobial therapy was administered. The patient achieved favorable recovery with no recurrence at 2-month follow-up. To our knowledge, this is the first reported case of *Aeromonas hydrophila* sepsis after combined endoscopic retrograde cholangiopancreatography and laparoscopic cholecystectomy. Personalized 1 h bundle implementation is vital to optimize prognosis in high-risk patients. This case also highlights the importance of strict adherence to endoscope reprocessing protocols, routine quality monitoring, and water quality management to minimize procedure-related infection risks.

## Introduction

Gallbladder stones combined with common bile duct stones are frequently encountered in elderly patients, and the standard minimally invasive management consists of Endoscopic Retrograde Cholangiopancreatography (ERCP) for stone extraction followed by laparoscopic cholecystectomy (LC) (1). Postoperative infectious complications remain a notable clinical concern despite the advantages of minimally invasive procedures (2). *Aeromonas hydrophila* (*A. hydrophila*) is a highly virulent pathogen that can induce life-threatening systemic infection (3), yet postoperative sepsis caused by this organism is extremely rare in patients undergoing biliary endoscopic and surgical interventions.

This case reports a rare *A. hydrophila* sepsis following combined ERCP and LC, which was rapidly rescued with the 1 h sepsis bundle. This report emphasizes the importance of early recognition of rare pathogenic infections, prompt implementation of standardized sepsis protocols, and strict infection control in elderly high-risk patients undergoing biliary endoscopic and surgical procedures.

## Case presentation

A 76-year-old married woman, with a history of hypertension and diabetes but no other medical or family history, presented with recurrent right upper quadrant pain for over a month. She exhibited mild tenderness and a positive Murphy’s sign. An MRI revealed a nodular filling defect in the distal common bile duct and mild dilation of the proximal duct, along with a thickened gallbladder wall and a nodular defect within the gallbladder. She was diagnosed with gallbladder stones and common bile duct stones. Given the patient’s advanced age and poor health, and after thorough discussion and considering their wishes, we opted for a staged treatment: first ERCP to remove the stones, then LC.

**Figure 1:**
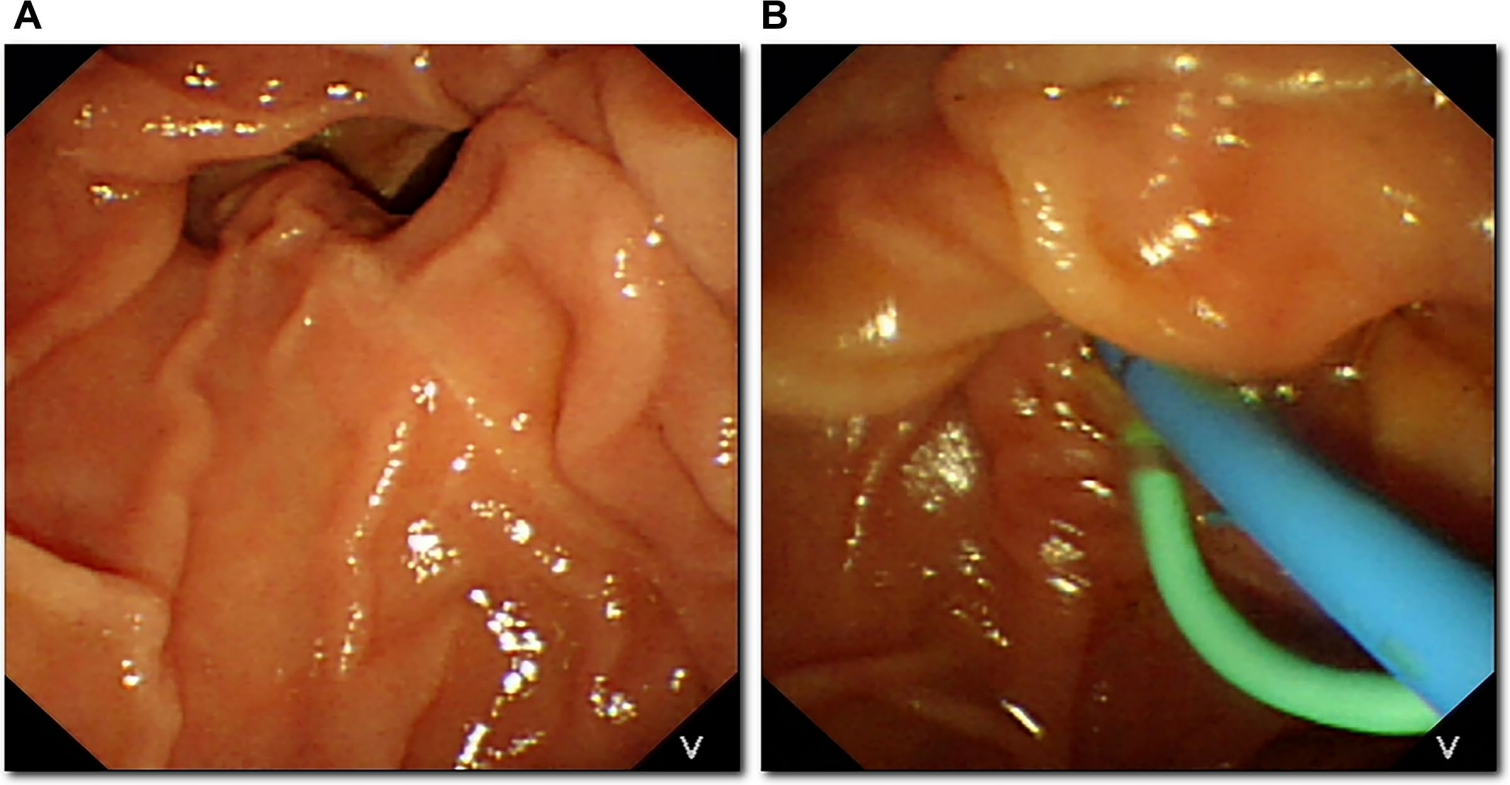
Illustration of intraoperative endoscopic retrograde cholangiopancreatography (ERCP) procedure. **(A)** ERCP showing normal mucosa of duodenal bulb and papilla region without instruments; **(B)** Endoscopic stent implantation under ERCP, blue indicates biliary stent and green indicates pancreatic duct stent.

On Hospital Day 1, the patient underwent ERCP under topical pharyngeal anesthesia. No prophylactic antibiotics were administered before or during the ERCP. Endoscopic sphincterotomy (EST) with a 0.3 cm incision on the duodenal papilla was performed prior to stone retrieval. Intraoperative contrast injection showed mild dilation of the common bile duct (approximately 0.9 cm in diameter), and a movable filling defect of 0.5 cm was found in the distal common bile duct. One black calculus and multiple muddy biliary sediments were extracted with a disposable stone basket, and no lithotripsy devices were utilized throughout the procedure. Initial selective biliary cannulation failed, and the guidewire accidentally entered the pancreatic duct. To reduce the risk of post-ERCP pancreatitis, a pancreatic duct stent and a biliary stent were simultaneously implanted for biliary decompression (Figures 1A,B).

On hospital day 4 (3 day after ERCP), the patient successfully underwent a LC under general anesthesia.

In the morning of Hospital Day 5 (1 day after LC), laboratory tests indicated mild abnormalities in high-sensitivity C-reactive (hs-CRP) protein and liver function markers, which were attributed to surgical stress. That afternoon, the patient acutely developed severe epigastric discomfort, accompanied by chills, high fever, and mild confusion. The vital signs were as follows: temperature 39.2 °C, pulse rate 117 bpm, respiratory rate 24 bpm, blood pressure 120/67 mmHg, and SpO_2_ 88%. Urgent laboratory results revealed a significant decrease in white blood cell (WBC) count from 8.37 × 10^9^/L to 1.14 × 10^9^/L within 10 h; the platelet count was 118 × 10^9^/L, procalcitonin (PCT) level was 0.343 ng/mL, and (hs-CRP) level was 141.75 mg/L. A chest and abdominal CT scan was performed, which ruled out intra-abdominal hemorrhage and abscesses and confirmed proper positioning of the biliary and pancreatic stents. The patient’s PaO_2_/FiO_2_ ratio was 303, the Glasgow Coma Scale score was 13, and the Sequential Organ Failure Assessment (SOFA) score was 2, with 1 point each for respiratory and nervous systems and 0 points for other organs. The baseline SOFA score before deterioration was 0.

To determine the cause of the altered mental status, a stepwise differential diagnosis was performed. Continuous postoperative analgesia with flurbiprofen axetil achieved adequate control of surgical incision pain. The new-onset severe upper abdominal pain was partially relieved after symptomatic treatment, whereas the altered mental status persisted, ruling out pain-induced mental disturbance. No central analgesics or sedatives were administered after LC, excluding drug-induced cerebral dysfunction. The patient remained fully conscious during previous febrile episodes with equivalent peak temperatures. Immediate combined physical and pharmacological cooling was initiated for the current fever, yet neurological abnormalities persisted despite a significant temperature reduction, eliminating isolated hyperthermia as the cause of neurological symptoms. The patient had no underlying central nervous system disorders, including dementia, cerebral infarction, or cerebral atrophy, and exhibited no age-related neurological vulnerability. The GCS score remained 13 throughout the clinical course, with no focal neurological deficits (hemiplegia, seizures, projectile vomiting, anisocoria) or signs of elevated intracranial pressure. Serial bedside neurological examinations excluded intracranial organic lesions, and thus CT or MRI was not performed. The patient’s mental status changes were in sync with significant rises in systemic inflammatory markers, which led us to associate her neurological dysfunction with sepsis.

Following a consultation with the Department of Critical Care Medicine, and based on clinical presentation, laboratory findings and the SOFA score, sepsis was diagnosed according to the Sepsis-3 criteria. The 1 h sepsis bundled care protocol was immediately initiated: arterial blood lactate was urgently measured at 1.6 mmol/L; blood cultures were collected within 30 min of sepsis identification, and the first dose of empirical meropenem (1 g IV every 8 h) was administered within 45 min; within 1 h, 1,000 mL of balanced salt solution was administered for moderate fluid resuscitation. The patient’s lactate levels were normal and hemodynamics were stable; therefore, the guideline-recommended standardized crystalloid resuscitation regimen of 30 mL/kg was not implemented. The patient’s blood pressure remained stable without the need for vasopressors, and other symptomatic supportive treatments were administered concurrently. At 6 h post-resuscitation, the lactate level was rechecked and found to be 1.3 mmol/L. In the early hours of Hospital Day 6 (Day 1 of confirmed sepsis), laboratory examinations yielded inflammatory markers including WBC count 12.3 × 10^9^/L, PCT 34.646 ng/mL and hs-CRP 171.28 mg/L. The drastically increased levels of these biomarkers indicated an ongoing infectious process.

**Figure 2:**
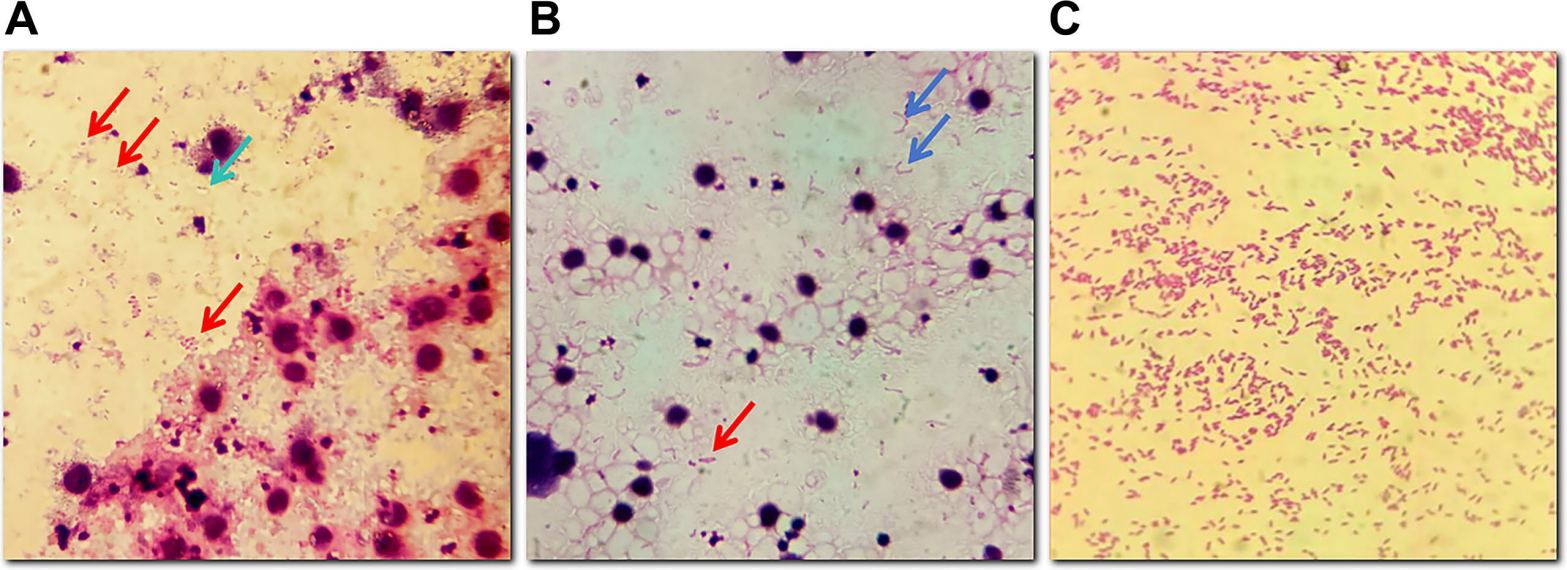
Gram staining of original blood smear and colony smear (2000× magnification). **(A)** Bacterial morphology in aerobic culture tube; red arrows indicate rod-shaped and coccobacillary bacteria, and blue arrows point to capsules, which assists in narrowing the differential diagnosis to virulent encapsulated pathogens and ruling out Gram-positive bacteria. **(B)** Bacterial morphology in anaerobic culture tube; red arrows show rod-shaped bacteria, and blue arrows mark filamentous bacteria, reflecting a pleomorphic stress response and suggesting a facultative anaerobe rather than a strict aerobe. **(C)** Morphology of colonies on smear after Gram staining (magnification, 2000×), confirming the purity of the clinical isolate, which serves as a prerequisite for reliable automated biochemical identification.

**Figure 3:**
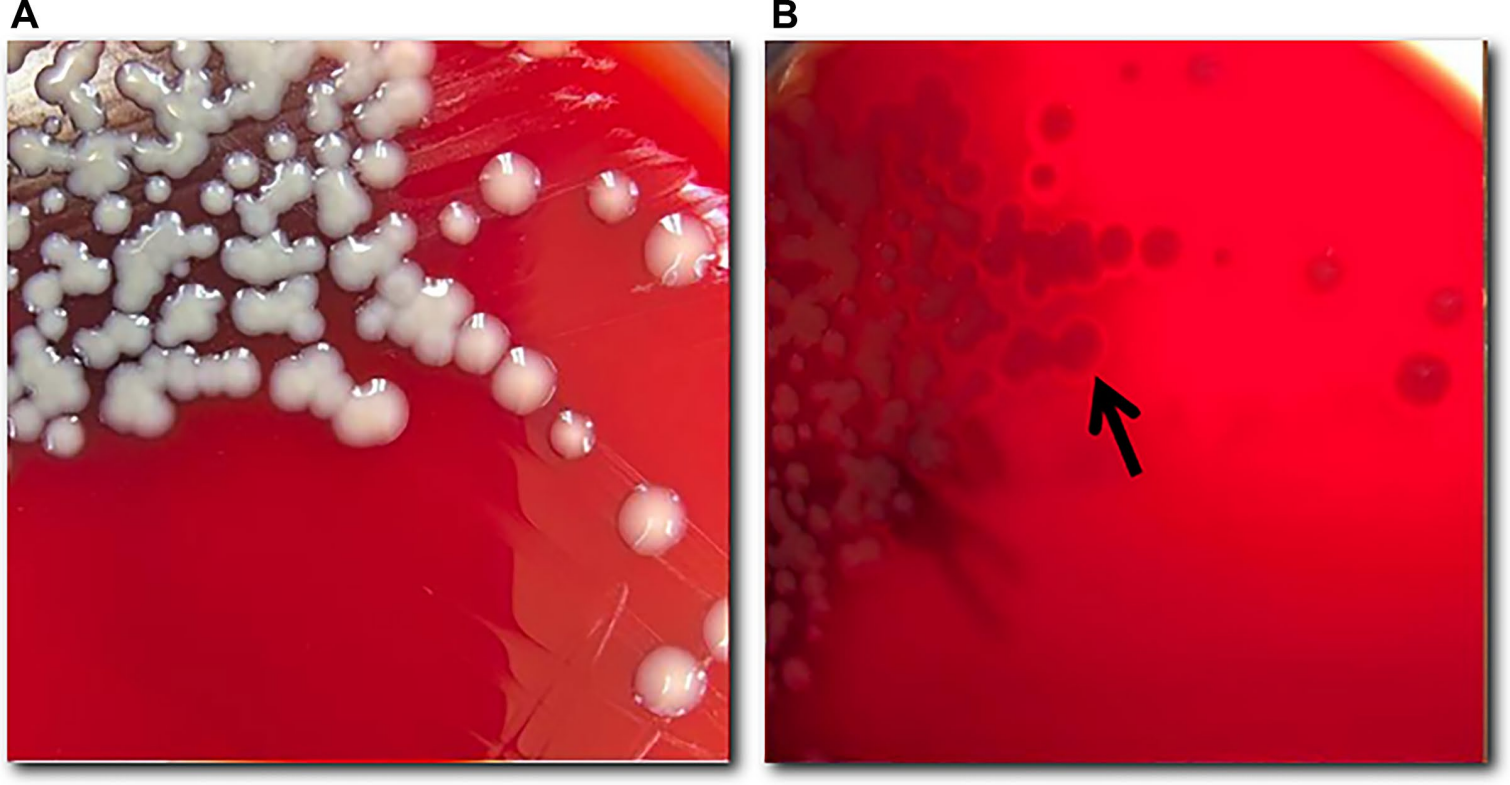
Colony morphology and β-hemolytic characteristics of the cultured isolate. **(A)** Colony morphology on Columbia blood agar after incubation at 35 °C for 24 h, showing gray, moist colonies that indicate the initial phenotypic characteristics of the isolate. **(B)** The arrow indicates a narrow zone of β-hemolysis, serving as a critical diagnostic marker that confirms exotoxin-producing pathogenicity; this strong hemolytic feature directly warrants subsequent confirmation via MALDI-TOF mass spectrometry.

Under the oil immersion objective, Gram-negative bacteria were detected in the patient’s blood smear after Gram staining. These bacteria exhibited varying morphologies: they were occasionally rod-shaped or coccobacillary with distinct, often asymmetrical capsules in aerobic conditions (Figure 2A), while in anaerobic conditions, they appeared filamentous (Figure 2B). When a smear of a pure culture was prepared and examined microscopically post-Gram staining, the bacteria consistently appeared as Gram-negative rods (Figure 2C). Blood agar plates inoculated with the blood specimen were incubated at 35 °C for 24 h, resulting in medium-sized, round, smooth, moist, convex, grayish-white colonies. These colonies displayed narrow *β*-hemolytic rings when observed against the light (Figures 3A,B). Further identification was conducted using the Clin-ToF-II time-of-flight mass spectrometry system, and based on the morphological characteristics of the colonies, the organism was conclusively identified as *A. hydrophila*. Antimicrobial susceptibility testing was conducted using broth microdilution, with results interpreted per the 2024 CLSI M45 standard. MIC values and susceptibility outcomes for all tested antibiotics are presented in Table 1.

**Table 1:** Antimicrobial susceptibility results of *A. hydrophila* isolates.

Antibacterial drugs	MIC value (μg/mL)	Susceptibility
Piperacillin/Tazobactam	≤4	S
Cefuroxime sodium	≤1	S
Cefoxitin	≤4	S
Ceftazidime	≤0.12	S
Ceftriaxone	≤0.25	S
Cefoperazone/Sulbactam	≤8	S
Cefepime	≤0.12	S
Imipenem	≤0.25	S
Amikacin	≤2	S
Levofloxacin	≤0.12	S
Tigecycline	≤0.5	S
Co-trimoxazole	≤20	S

MIC, minimum inhibitory concentration; S, susceptible; I, intermediate; R, resistant. The susceptibility of *A. hydrophila* to different antibacterial agents was determined and interpreted in accordance with the Clinical and Laboratory Standards Institute (CLSI) guidelines.

**Figure 4:**
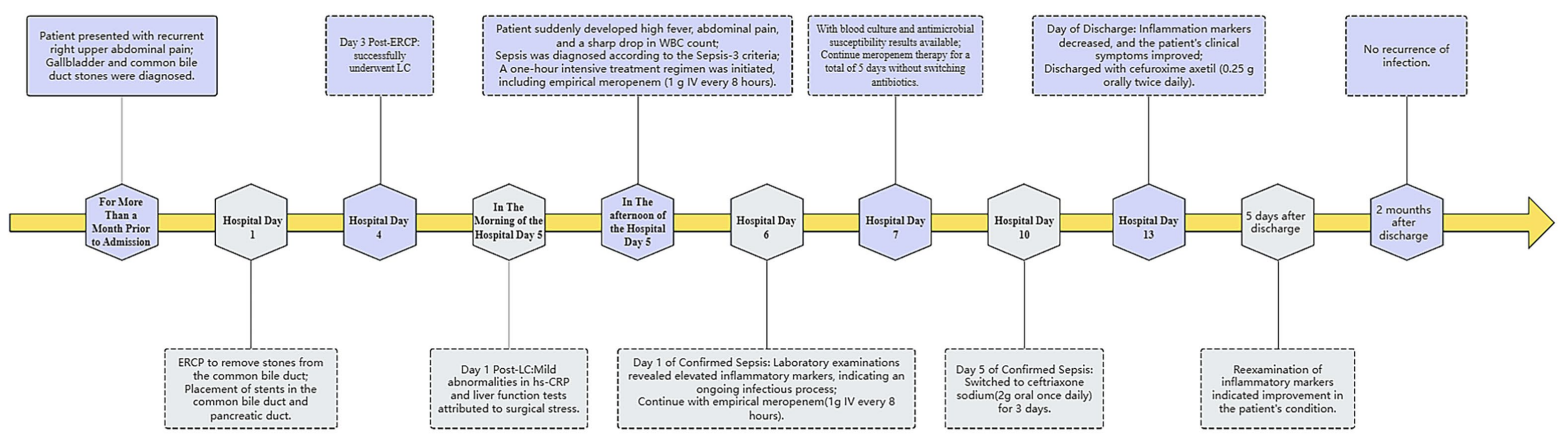
Flow chart of diagnosis and treatment process of the patient.

On Hospital Day 7 (Day 2 of confirmed sepsis), with blood culture and antimicrobial susceptibility results were available, the patient’s inflammatory markers were as follows: WBC count 10.37 × 10^9^/L, hs-CRP 175.81 mg/L, and PCT 18.29 ng/mL. Therefore, we did not switch antibiotics immediately and continued meropenem (1 g IV every 8 h), with a total treatment course of 5 days. Blood tests conducted on Hospital Day 10 (Day 5 of confirmed sepsis) showed a WBC count of 3.11 × 10^9^/L, hs-CRP of 43.08 mg/L, and PCT of 3.159 ng/mL. Based on the blood culture and susceptibility test results as well as consultation opinions from the clinical pharmacy department, the anti-infective regimen was adjusted to ceftriaxone sodium (2 g IV once daily) for standardized targeted therapy lasting 3 days. Repeat blood tests were performed on Hospital Day 13 (Day of discharge), revealing a WBC count of 2.56 × 10^9^/L, hs-CRP of 27.04 mg/L, and PCT of 1.638 ng/mL. The patient’s clinical status improved, and she was discharged with sequential cefuroxime axetil tablets prescribed upon discharge (0.25 g orally twice daily). Routine blood tests and inflammatory biomarker reexamination were conducted 5 days after discharge, demonstrating a WBC count of 6.45 × 10^9^/L, hs-CRP of 4.23 mg/L, and PCT of 0.28 ng/mL. No infectious recurrence was observed during the 2-month follow-up period. The patient’s clinical diagnosis and treatment timeline is shown in the figure (Figure 4).

## Discussion

ERCP is an indispensable therapeutic intervention with well-established indications for a variety of complex pancreaticobiliary diseases, including cholecystolithiasis complicated by choledocholithiasis (4). Although ERCP provides deterministic efficacy, it carries a notable risk of postoperative complications such as pancreatitis, hemorrhage, perforation, and biliary tract infection, which in severe cases can induce life-threatening systemic sepsis. Sepsis refers to life-threatening organ dysfunction induced by dysregulated host responses to infection, and early targeted therapy improves patient prognosis (5). The 2021 Surviving Sepsis Campaign (SSC) guidelines set stratified timelines for antimicrobial administration: antibiotics should be initiated within 1 h for patients with septic shock, while a 3 h window for infection evaluation is available for those without shock (6). Although this patient did not develop septic shock, he presented prominent high-risk clinical manifestations after two biliary surgeries, so we promptly implemented the 1 h sepsis bundle protocol.

Following two invasive biliary procedures, the patient acutely developed a high fever, abdominal pain, and mild altered mental status. An urgent peripheral blood test indicated a significant decline in white blood cell count. While an elevated white blood cell count is a classic laboratory indicator of sepsis, leukopenia suggests severe endotoxemia and serves as a biomarker of poor prognosis (7). Clinically, a severe invasive bacterial infection was strongly suspected. According to the 2021 SSC guidelines, patients without septic shock may be allowed a 3 h window for infection assessment. However, after a thorough risk stratification assessment, this patient was identified as having multiple significant high-risk factors. Consequently, delayed observation was deemed inappropriate, and all core procedures of the 1 h sepsis bundle were executed within the specified timeframe: lactate testing, blood culture collection, empirical administration of meropenem for antimicrobial therapy, and individualized fluid resuscitation.

*A. hydrophila* was identified 48 h post blood culture collection, accompanied by a complete minimum inhibitory concentration (MIC) susceptibility report. Although meropenem was not included in the susceptibility testing profile, imipenem—a carbapenem belonging to the same class—exhibited excellent *in vitro* antimicrobial activity against this strain. The initial selection of meropenem was informed by two primary considerations. Firstly, following the collection of blood cultures, the pathogen identification and susceptibility results were not immediately available. Given the patient’s critical condition and the strong suspicion of postoperative biliary tract infection, carbapenems were deemed the most appropriate broad-spectrum empirical agents for covering potential pathogens. Secondly, at the time the susceptibility results were reported, the patient’s inflammatory markers remained significantly elevated, indicating that the infection was not yet controlled. The strain’s high susceptibility to imipenem suggests an inherent sensitivity to carbapenems. Given that meropenem and imipenem possess similar antimicrobial spectra and bactericidal activity, it can be inferred that meropenem is effective against the causative pathogen in this case. At this juncture, transitioning prematurely to an antibiotic of lower potency would likely exacerbate the infection. Consequently, meropenem monotherapy was maintained for a total treatment duration of 5 days. Following this period, the patient exhibited significant improvement in both symptoms and inflammatory markers. After consultation with a clinical pharmacist, the treatment regimen was adjusted to ceftriaxone sodium. This modification was informed by the results of antibiotic susceptibility testing and the pharmacological properties of ceftriaxone sodium, which include effective penetration of biliary tract tissues. Additionally, this adjustment aimed to minimize carbapenem exposure and mitigate the risk of developing bacterial resistance. The patient’s condition continued to improve with the subsequent treatment course. The 2026 edition of the Surviving Sepsis Campaign (SSC) guidelines mandates the initiation of antibiotics within 1 h for patients with sepsis, with blood cultures obtained prior to administration (8). The management of this case was in full compliance with these guidelines.

To identify the source of infection in this case, a systematic analysis of five potential transmission routes is necessary. *A. hydrophil* is a common microorganism found in freshwater environments. During ERCP, the endoscope and its related instruments come into direct contact with water during the reprocessing and rinsing stages, which presents a plausible biological pathway for the introduction of exogenous bacteria. Given that the patient had no prior exposure to external water sources, exogenous contamination emerges as the most likely cause. Several studies have documented outbreaks of multidrug-resistant bacterial infections following ERCP, attributed to non-standard endoscope reprocessing procedures, contaminated instruments, or contaminated surfaces (9, 10). Although papillotomy and double-stent placement compromise the Oddi sphincter barrier, *A. hydrophil* predominantly inhabits freshwater environments and is not part of the normal human intestinal flora; thus, endogenous reflux alone cannot sufficiently account for this infection. CT scans demonstrated the absence of abdominal abscesses or surgical site infections, yet the patient developed sepsis within 24 h postoperatively. This suggests that laparoscopic cholecystectomy is unlikely to be the primary source of infection, as transient bacteremia is typically rapidly cleared by the immune system. Bacteremia resulting from biliary obstruction or invasive procedures necessitates prior colonization of the biliary tract with *A. hydrophil*, with colonization sources categorized as either exogenous or endogenous. Considering the patient’s extended hospitalization and absence of relevant exposure history, community-acquired or environmental infection can largely be excluded. In conclusion, exogenous contamination associated with ERCP emerges as the most probable initial source of infection. However, due to the lack of confirmatory microbiological evidence—such as cultures from the endoscope, flushing water, or aspirated bile—the precise source of infection remains indeterminate.

*A. hydrophil* is a Gram-negative bacillus commonly present in various aquatic environments, including potable water, wastewater, sewage, and a range of food products (11). This bacterium is recognized as an emerging pathogen with the potential to cause gastroenteritis, skin infections, and systemic illnesses such as peritonitis, meningitis, and bacteremia (12). It is considered a conditionally pathogenic microorganism, capable of infecting immunocompromised individuals or entering the human body through open wounds exposed to contaminated water. In the current literature, instances of *A. hydrophil* bacteremia following ERCP are exceedingly rare. The patient had no documented history of exposure to contaminated water outside the hospital, nor was there any epidemiological evidence indicating exposure to a contaminated community environment. The timing of sepsis onset could only indirectly suggest a link between the pathogen and the preceding ERCP procedure. Nevertheless, the healthcare facility did not conduct a comprehensive series of microbiological traceback investigations, including bacterial cultures of the endoscope, irrigation fluid, environmental surfaces, and biliary aspirates. Moreover, the disinfection process of the endoscope was not verified, nor was any investigation into a potential nosocomial outbreak conducted. Consequently, the contamination of the ERCP instruments or bacterial colonization associated with the stent remains a biologically plausible hypothesis for the infection, yet it has not been confirmed. Thus, the precise route of contamination in this case cannot be definitively ascertained at this time.

Nevertheless, published studies have well documented clear biological mechanisms underlying ERCP-related bacteremia transmission. Over the last decade, ERCP-related bacteremia outbreaks caused by bacteria including carbapenem-resistant *Escherichia coli*, susceptible *E. coli*, and *Pseudomonas aeruginosa* have increased. Key factors include poor reprocessing, biofilm formation, design flaws, and endoscope damage, with biofilm formation being the most critical (13). Biofilms are protective protein matrices created by bacteria to shield against antibiotics and disinfectants. In favorable conditions, bacteria in biofilms can multiply, detach, and become free-floating, potentially spreading infections during procedures and causing endoscope-related cross-infections (14, 15). Clinicians should maintain high suspicion of *A. hydrophila* -mediated sepsis in critically ill patients with abrupt leukopenia and a history of ERCP. Prompt initiation of the 1 h sepsis bundle plus empirical antibiotics active against *Aeromonas* spp. can improve patient clinical outcomes.

According to the World Health Organization (WHO) guidelines on infection control and medical device reprocessing, the implementation of standardized prevention protocols is essential for endoscopic procedures. Under the traditional Spaulding classification system adopted by the WHO, duodenoscopes are categorized as semi-critical devices because they are designed to contact intact mucosa, thereby requiring high-level disinfection (HLD) at a minimum. However, in advanced interventions such as ERCP, these complex devices and their accessories routinely interact with broken mucosa or enter sterile body cavities. This clinical reality has prompted a significant paradigm shift in professional consensus, with organizations like the SGNA actively advocating for the reclassification of duodenoscopes as critical devices that mandate absolute sterilization rather than mere HLD. It is imperative for healthcare facilities to establish robust quality control systems to oversee reprocessing activities, ensure complete traceability, and provide comprehensive staff training (16, 17). Given the isolation of *A. hydrophila* in this instance, meticulous management of rinse water is crucial to prevent similar infections. Despite adherence to these protocols, the formation of biofilms within the complex lumens of endoscopes cannot be entirely precluded. Although defective reprocessing has not been confirmed in this case, it remains a potential transmission route. Clinicians are advised to consistently adhere to WHO standards, with a particular emphasis on manual brushing, thorough drying of lumens, and vigilant water monitoring to mitigate the risk of waterborne nosocomial infections.

## Conclusion

We present the first reported case of sepsis induced by *A. hydrophila* after combined ERCP and laparoscopic cholecystectomy. The patient developed typical manifestations of sepsis accompanied by an obvious drop in white blood cell count. For high-risk patients receiving biliary tract procedures, timely implementation of individualized 1 h sepsis bundle is vital to optimize prognosis. This case also reminds clinicians to strictly comply with standardized endoscope reprocessing specifications, conduct regular quality surveillance, and strengthen rinse water management to lower the risks of procedure-associated infections.

## Data Availability

The original contributions presented in the study are included in the article/supplementary material, further inquiries can be directed to the corresponding authors.
